# Gadolinium Chloride Restores the Function of the Gap Junctional Intercellular Communication between Hepatocytes in a Liver Injury

**DOI:** 10.3390/ijms20153748

**Published:** 2019-07-31

**Authors:** Le Yang, Chengbin Dong, Lei Tian, Xiaofang Ji, Lin Yang, Liying Li

**Affiliations:** Department of Cell Biology, Municipal Laboratory for Liver Protection and Regulation of Regeneration, Capital Medical University, Beijing 100069, China

**Keywords:** gap junctional intercellular communication, connexin, liver injury, hepatocyte, gadolinium chloride

## Abstract

**Background:** Gadolinium chloride (GdCl3) has been reported to attenuate liver injury caused by a variety of toxicants. Gap junctional intercellular communication (GJIC) is thought to be essential in controlling liver homeostasis and pathology. Here we evaluate the effects of GdCl3 on functional GJIC and connexin expression in mouse models and primary hepatocytes. **Methods:** Mice were administered GdCl3 intraperitoneally the day before a carbon tetrachloride (CCl4) injection or bile duct ligation (BDL) operation. Primary hepatocytes were treated with CCl4 or lipopolysaccharides (LPS), with or without GdCl3. A scrape loading/dye transfer assay was performed to assess the GJIC function. The expression of connexins was examined by real-time reverse transcription polymerase chain reaction (RT-PCR), western blot and immunofluorescent staining. **Results:** CCl4 treatment or the BDL operation led to the dysfunction of GJIC and a down-regulation of Cx32 and Cx26 in injured liver. GdCl3 administration restored GJIC function between hepatocytes by facilitating the transfer of fluorescent dye from one cell into adjacent cells via GJIC, and markedly prevented the decrease of Cx32 and Cx26 in injured liver. In primary hepatocytes, CCl4 or LPS treatment induced an obvious decline of Cx32 and Cx26, whereas GdCl3 pretreatment prevented the down-regulation of connexins. In vivo GdCl3 protected hepatocytes and attenuated the liver inflammation and fibrosis in liver injury mouse models. **Conclusion:** GdCl3 administration protects functional GJIC between hepatocytes, and prevents the decrease of connexin proteins at mRNA and protein levels during liver injury, leading to the alleviation of chronic liver injury.

## 1. Introduction

Gap junctional intercellular communication (GJIC) is mediated by gap junctions, which are transmembrane channels linking neighboring cells and providing the only pathway to transfer small hydrophilic cytoplasmic metabolites less than 1000 Dalton, growth modulators and second messengers between the adjacent cells [1,2]. Gap junctions are composed of two hemi-channels, and each hemi-channel consists of six connexin protein units. At present, 21 connexin proteins have been identified in humans, while 20 connexins have been characterized in rodents. They all share a similar structure consisting of four membrane-spanning domains, two extracellular loops, a cytoplasmic loop, a cytosolic N-terminal and C-terminal regions [3]. Connexins are named after their molecular weight. In mouse liver, hepatocytes predominantly express connexin32 (Cx32), and to a less extent connexin26 (Cx26) in the gap junctional plaque [4,5]. Most tissues express more than one connexin type that can be regulated by hormones, growth factors and proinflammatory mediators, thus ensuring a fine-tuned regulation of GJIC [6,7]. 

GJIC has long been considered to play an important role in controlling tissue homeostasis and the cellular life cycle, ranging from cell growth to cell death [8]. In liver, the establishment of a well-orchestrated GJIC network between hepatocytes has been demonstrated numerous times as a prerequisite for the appropriate performance of hepatic functionality [9]. Several hepatocyte-specific functions, including albumin secretion, ammonia detoxification, glycogenolysis and bile secretion, require the presence of these junctions [10]. In addition, GJIC acts as major gatekeepers in the control of liver cell death and proliferation [11,12,13]. Hepatic connexin expression patterns undergo marked changes during chronic liver disease, such as liver fibrosis and cirrhosis, as well as during acute liver injury [14]. For example, it has been reported that there was a reduction in hepatic Cx32 content in carbon tetrachloride (CCl_4_)-induced chronic [15] and acute [16] liver injury. Cx32 plasma membrane immunofluorescent, protein and mRNA levels also decreased markedly after bile duct ligation (BDL) [17]. In addition, there was a switch in RNA and protein production from Cx32 and Cx26 to Cx43 upon acetaminophen intoxication in rodents [18]. However, it is required for connexins to traffic to the membrane and to correctly assemble into gap junction plaques to form functional gap junction channels, and previous reports have suggested that some connexins do not contribute to the cell-cell communication [19], thus it is more important to examine the changes of GJIC at the functional level during liver injury and tissue restoration.

The lanthanide trivalent gadolinium is widely applied to much medical practice. For example, it has been used in diagnosis as a magnetic resonance imaging contrast agent [20]. It also has therapeutic applications for many diseases, including hypertension and cardiac problems, Duchenne muscular dystrophy, and rheumatoid arthritis [21,22,23]. An interesting potential application of gadolinium chloride (GdCl_3_) is its contribution to the reduction of liver injury. It has been shown to protect against liver damage caused by a variety of toxicants including ethanol, dimethylnitrosamine, CCl_4_ and cadmium [24,25,26]. Now, even though some reports suggest that the hepatoprotective effect of GdCl_3_ was due to the inactivation and destruction of Kupffer cells (KCs) [27], GdCl_3_ may cause additional effects which lead to a reduction in liver injury. For example, Harstad and Klaassen suggest that GdCl_3_ may increase hepatic metallothionein, and thus protect the liver from cadmium-induced liver injury [28]. Moreover, Hirasawa et al. demonstrate that GdCl_3_ can penetrate into hepatocytes and protect isolated hepatocytes in vitro by affecting cytochrome P450 [29]. Interestingly, GdCl_3_ has been shown to affect the function of gap junction hemi-channels in *Xenopus laevis* (African Claw Frog) oocytes as a chloride-channel blocker [30]. Based on this, we hypothesized that the protective effects of GdCl_3_ could be mediated by the alteration of GJIC in chronic liver injury. 

The aim of this study is to investigate the modulation of GdCl_3_ on GJIC both in mouse models of chronic liver injury and primary cultured hepatocytes by examining GJIC at the functional, mRNA, and protein levels, as well as the intracellular localization of connexins. The present study clearly shows that GdCl_3_ acts to protect functional GJIC between hepatocytes and prevents the decrease of connexin proteins Cx32 and Cx26 at mRNA and protein levels in liver injury mice and primary hepatocytes, which provides new insights into the effects of GdCl_3_ during liver injury.

## 2. Results

### 2.1. Cx32 and Cx26 Were Markedly Down-Regulated in Injured Liver

**Figure 1:**
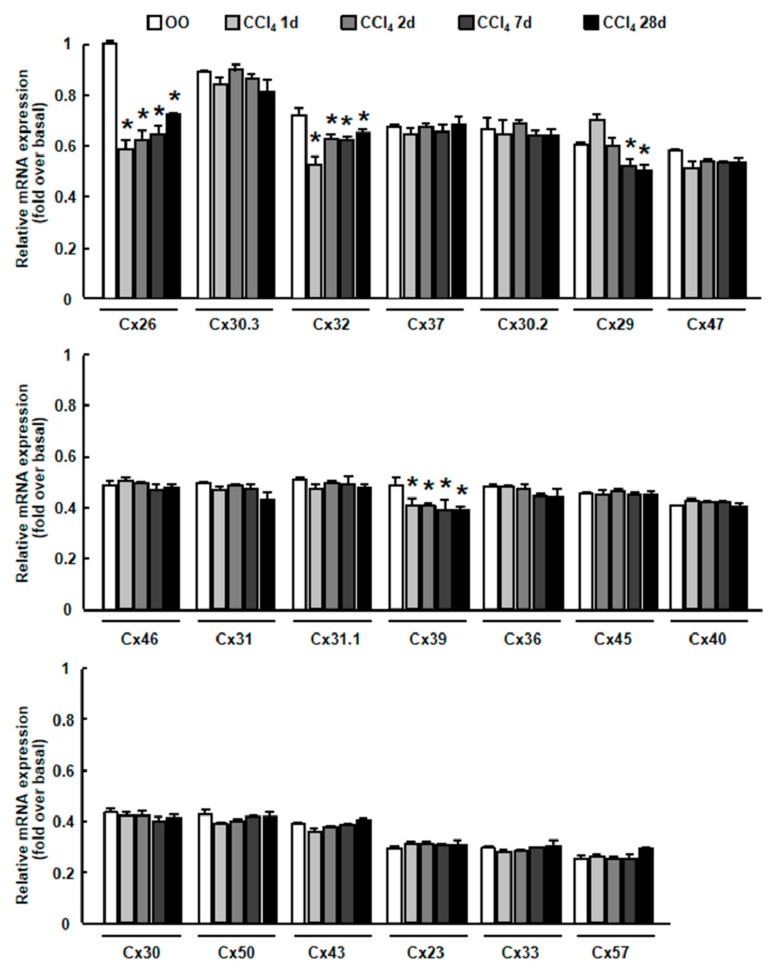
The expression pattern of multiple connexins in carbon tetrachloride (CCl_4_)-injured mouse liver. The mRNA expression of different connexins was examined by a quantitative real-time reverse transcription polymerase chain reaction (qRT-PCR) in liver injury mice induced by CCl_4_. Data are presented as the mean ± SEM, and were analyzed by Student’s t-test for analysis of variance. * *p* < 0.05 *vs*. OO-treated mice.

Mice received intraperitoneal injection of CCl_4_ for different times to induce rodent liver injury models. The expression pattern of multiple connexins was investigated by real-time RT-PCR in injured livers. Among the 20 connexins which have been characterized in rodents [3], two of them (Cx32, Cx26) were markedly down-regulated at the mRNA levels throughout the entire stage of liver injury (from 1 day to 28 days’ CCl_4_ treatment) (Figure 1). Cx32 and Cx26 had been reported to be two important connexin proteins mainly expressing on hepatocytes [4]; Cx32 and Cx26 had higher abundance compared to Cx39. Based on this, we focused on the critical role of Cx32 and Cx26 in liver injury mouse models and primary hepatocytes in the following studies.

**Figure 2:**
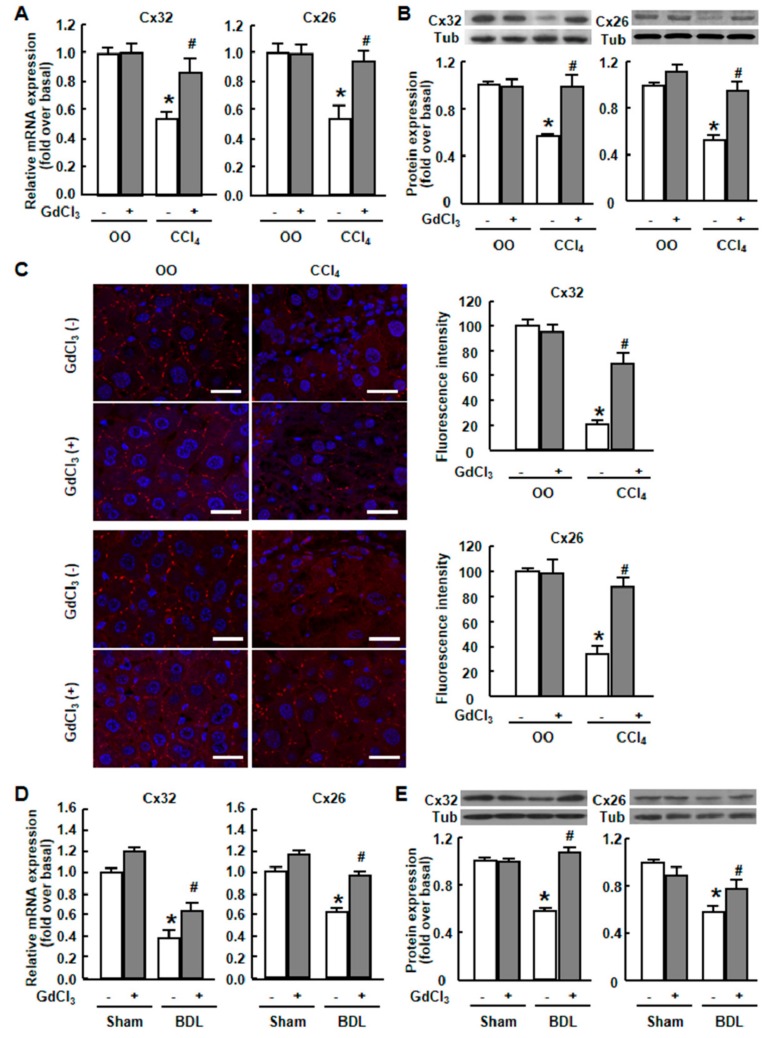
Cx32 and Cx26 expression in injured liver tissue with or without gadolinium chloride (GdCl_3_) administration. Four weeks after GdCl_3_ administration in CCl_4_-treated mice or two weeks after GdCl_3_ administration in bile duct ligation (BDL) mice, liver tissue was collected. Immuno-fluorescence staining, western blot and qRT-PCR analysis for the expression of Cx32 and Cx26 were performed. (**A**) Cx32 and Cx26 mRNA expression in CCl_4_-injured liver. (**B**) Cx32 and Cx26 protein expression in CCl_4_-injured liver. (**C**) Immunofluorescence staining for Cx32 and Cx26 in OO-treated, CCl_4_-treated, GdCl_3_ plus OO-treated and GdCl_3_ plus CCl_4_-treated liver. Scale bars, 20 μm. (**D**) Cx32 and Cx26 mRNA expression in BDL-injured liver. (**E**) Cx32 and Cx26 protein expression in BDL-injured liver. Typical autoradiograms are shown. Expression of tubulin was checked to correct for variations in protein loading and transfer. All results were confirmed in three independent experiments. Data are presented as the mean ± SEM, and were analyzed by ANOVA for analysis of variance. * *p* < 0.05 *vs*. OO-treated mice. # *p* < 0.05 *vs*. CCl_4_-treated mice without GdCl_3_ administration.

Real-time RT-PCR showed that the mRNA levels of Cx32 and Cx26 were significantly decreased to 54% and 53%, respectively, of normal levels after CCl_4_ treatment (Figure 2A). Western blot was performed to examine the protein expression of two major gap junction proteins in mouse liver, Cx32 and Cx26. In CCl_4_-treated mice, the protein content of Cx32 and Cx26 was markedly reduced to 57% and 52% of the normal levels, respectively (Figure 2B). We then examined their expression and localizations by immunofluorescence staining. Cx32 and Cx26 plaques were uniformly distributed in the plasma membrane throughout the liver tissue in normal mice (Figure 2C). 

After CCl_4_ treatment, Cx32- and Cx26-positive plaques per hepatocyte decreased in a non-uniform manner, and the reduction was very clear in the centrilobular region of the liver, which was consistent with the regional impairment of liver (Figure 2C). Similar decreased expression of Cx32 and Cx26 was observed in BDL-treated mouse liver (Figure 2D–E). These results demonstrated that liver injury caused a decrease of Cx32 and Cx26 both in mRNA and protein levels in mouse liver.

### 2.2. GdCl_3_ Prevented the Down-Regulation of Connexins and Restored the Cx32/Cx26 Plaques in Injured Mouse Liver

Recently GdCl_3_ has gotten more and more attention for its contribution to the attenuation of liver injury. Although several studies have linked the hepatoprotective effects of GdCl_3_ with the depletion of KCs [27], the underlying mechanisms are still unclear. To examine the effect of GdCl_3_ in vivo, we injected GdCl_3_ intraperitoneally into mice the day before the CCl_4_ administration or BDL operation. Real-time RT-PCR showed that the mRNA levels of Cx32 and Cx26, which were significantly decreased after CCl_4_ treatment, returned to 87% and 94% of normal levels, respectively, after the administration of GdCl_3_ (Figure 2A). In line with this, the results of western blot analysis showed that Cx32 and Cx26 protein levels were recovered almost to the normal levels after the administration of GdCl_3_ in CCl_4_-treated mice (98% and 95% of the normal levels, respectively) (Figure 2B). Such results were proportional to that observed by the immunofluorescence staining, showing that pretreatment with GdCl_3_ significantly prevented the reduction of Cx32 and Cx26 plaques in the liver, compared with CCl_4_-treated mice, and did not affect their localizations in the plasma membrane (Figure 2E). GdCl_3_ treatment alone had no effect on Cx32 or Cx26 expression in normal mice (Figure 2C). Similar phenomena were observed in BDL-treated mice (Figure 2D–E). Taken together, these results suggested that GdCl_3_ prevented the CCl_4_- or BDL-induced down-regulation of Cx32 and Cx26 in mouse liver. 

### 2.3. GdCl_3_ Restored the Functional GJIC in Injured Liver of CCl_4_-Treated Mice

**Figure 3:**
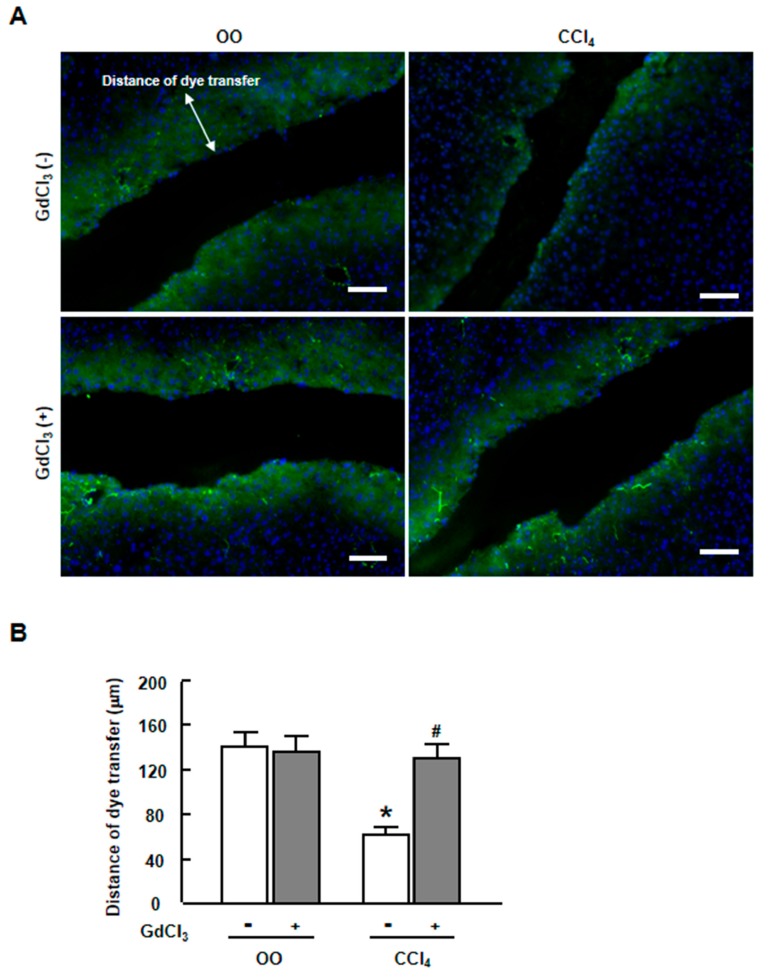
The gap junctional intercellular communication (GJIC) function in CCl_4_-treated mice with or without GdCl_3_ administration. (**A**) Digital images of Lucifer yellow dye transfer in liver treated with OO, CCl_4_, GdCl_3_ plus OO, GdCl_3_ plus CCl_4_. Scale bars, 100 μm. (**B**) GJIC function and integrity was determined by quantifying the distance of the Lucifer yellow dye transfer from the incision. Data are presented as the mean ± SEM and were analyzed by ANOVA for the analysis of variance. * *p* < 0.05 *vs*. OO-treated mice. # *p* < 0.05 *vs*. CCl_4_-treated mice without GdCl_3_ administration.

Next, we used scrape loading/dye transfer assay, applying Lucifer yellow, which is a kind of fluorescent dye that transfers through the gap junction channel, to examine hepatic GJIC function after CCl_4_-induced liver injury. The distance of the Lucifer yellow dye transfer from the incision was measured to evaluate the integrity and function of GJIC. As shown in Figure 3A,B the dye spread quite well in normal liver with a long distance from the incision. In contrast, the movement of Lucifer yellow dye across gap junctions was impaired after an administration of CCl_4_ with a markedly shorter distance, indicating the dysfunction of GJIC. In contrast, GdCl_3_ treatment apparently facilitated dye transfer compared with CCl_4_-treated mice, suggesting the restoration of functional GJIC (Figure 3A,B). GdCl_3_ treatment alone had no effect on dye transfer in normal mice (Figure 3A,B). These results demonstrated that GdCl_3_ might protect against the dysfunction of GJIC between hepatocytes in CCl_4_-treated murine liver. 

### 2.4. GdCl_3_ Prevented the Down-Regulation of Cx32 and Cx26 Induced by CCl_4_ or LPS in Primary Hepatocytes

**Figure 4:**
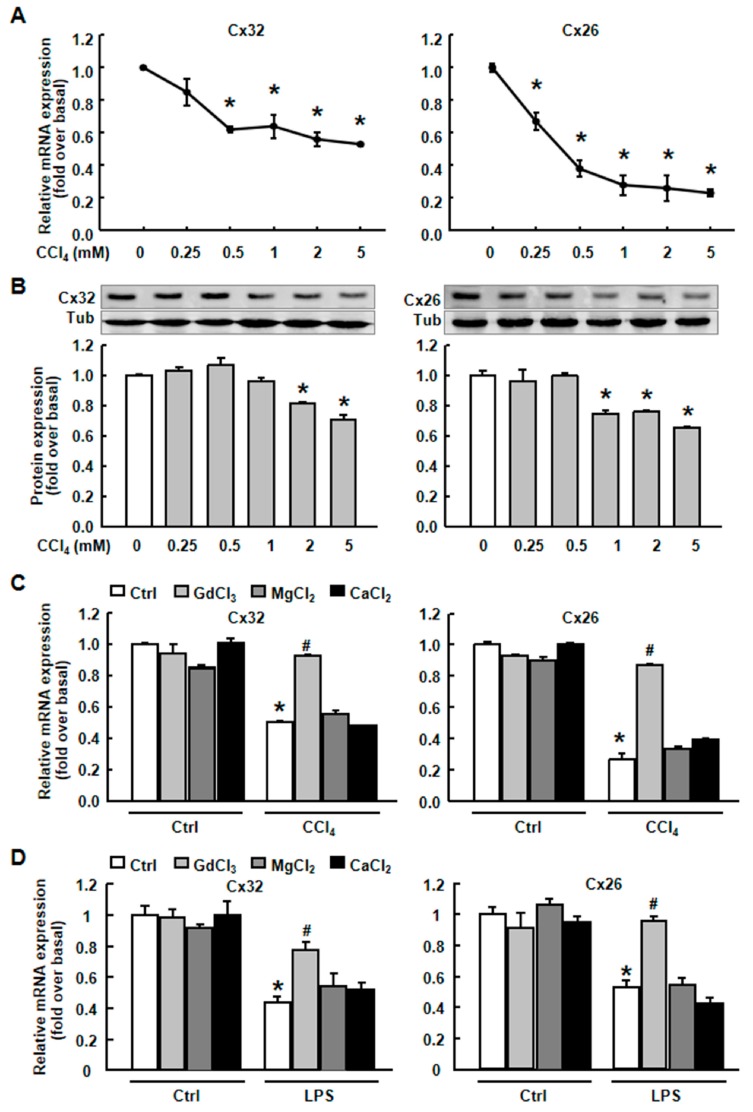
Cx32 and Cx26 expression in CCl_4_- or lipopolysaccharide (LPS)-injured primary hepatocytes with or without GdCl_3_ pretreatment. The expression of Cx32 and Cx26 mRNA (**A**) and protein (**B**) in primary hepatocytes which were treated with CCl_4_ of different concentrations. Cx32 and Cx26 mRNA expression in response to CCl_4_ (**C**) or LPS (**D**) with the pre-treatment of 200 μM GdCl_3_, 1 mM MgCl_2_ or 1 mM CaCl_2_ for 1 h, following 2 h of CCl_4_ or LPS treatment. All results were confirmed in three independent experiments. Data are presented as the mean ± SEM, and were analyzed by ANOVA for analysis of variance. * *p* < 0.05 *vs.* Ctrl. # *p* < 0.05 *vs.* CCl_4_-treated hepatocytes without GdCl_3_ administration.

To verify the down-regulation of the gap junction proteins in vitro, we treated primary hepatocytes with CCl_4_ or LPS to mimic the in vivo liver injury. As expected, the mRNA (Figure 4A) and protein levels (Figure 4B) of Cx32 and Cx26 were dramatically decreased after CCl_4_ treatment in a dose-dependent manner in primary hepatocytes. GdCl_3_ prevented the decreased mRNA expression of Cx32 and Cx26 induced by CCl_4_ or LPS, while MgCl_2_ or CaCl_2_, which has a similar biophysical character with GdCl_3_, had no such effect (Figure 4C,D). 

**Figure 5:**
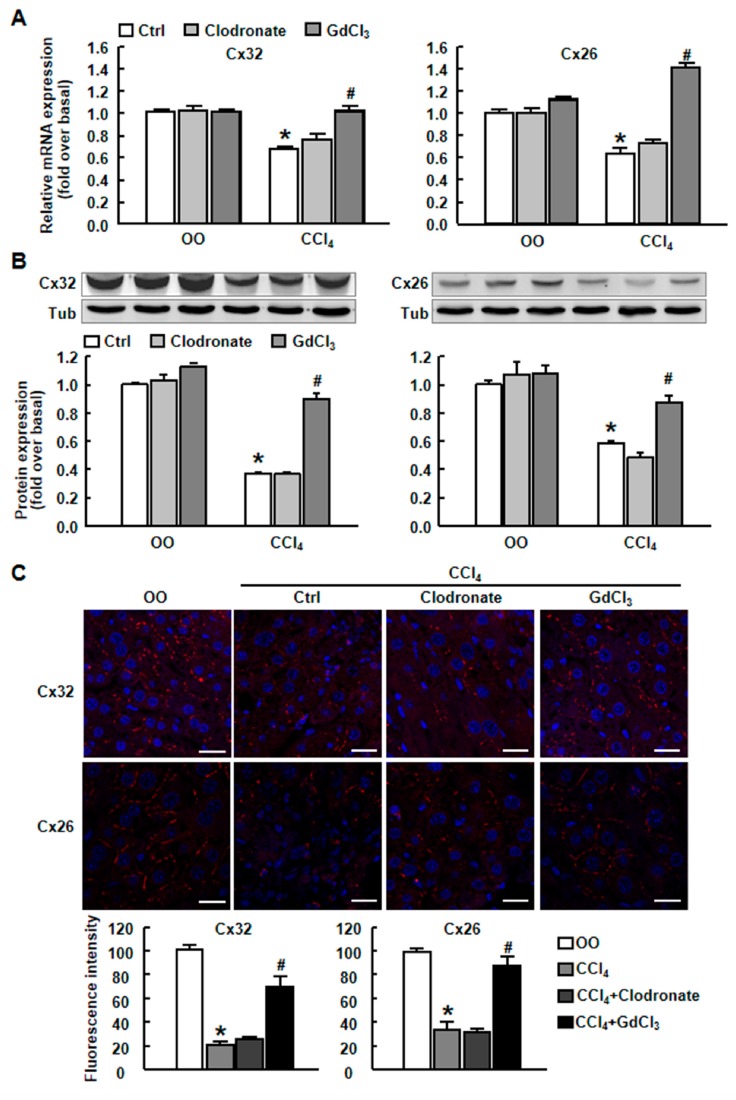
The expression of Cx32 and Cx26 in CCl_4_-treated liver tissue with or without clodronate administration. Clodronate liposome (10 L/g B.W.) injection was performed once 72 h before CCl_4_ treatment to delete Kupffer cells. The qRT-PCR, western blot analysis and immunofluorescence staining for the expression of Cx32 and Cx26 were performed. (**A**) Cx32 and Cx26 mRNA expression in injured liver. (**B**) Cx32 and Cx26 protein expression in injured liver. Typical autoradiograms are shown. Expression of tubulin was checked to correct for variations in protein loading and transfer. (**C**) Immunofluorescence staining for Cx32 and Cx26 in injured liver. Scale bars, 100 μm. All results were confirmed in three independent experiments. Data are presented as the mean ± SEM, and were analyzed by ANOVA for analysis of variance. * *p* < 0.05 *vs.* Ctrl. # *p* < 0.05 *vs.* CCl_4_-treated mice alone.

We used another Kupffer cell depletor Clodronate, and evaluated its effect on Cx32 and Cx26 expression in vivo, showing that GdCl_3_ but not Clodronate could rescue connexin expression in CCl_4_-treated mice, further indicating the specific action of GdCl_3_ on connexin expression and functional GJIC during liver injury, which is independent of KC depletion (Figure 5A–C). 

**Figure 6:**
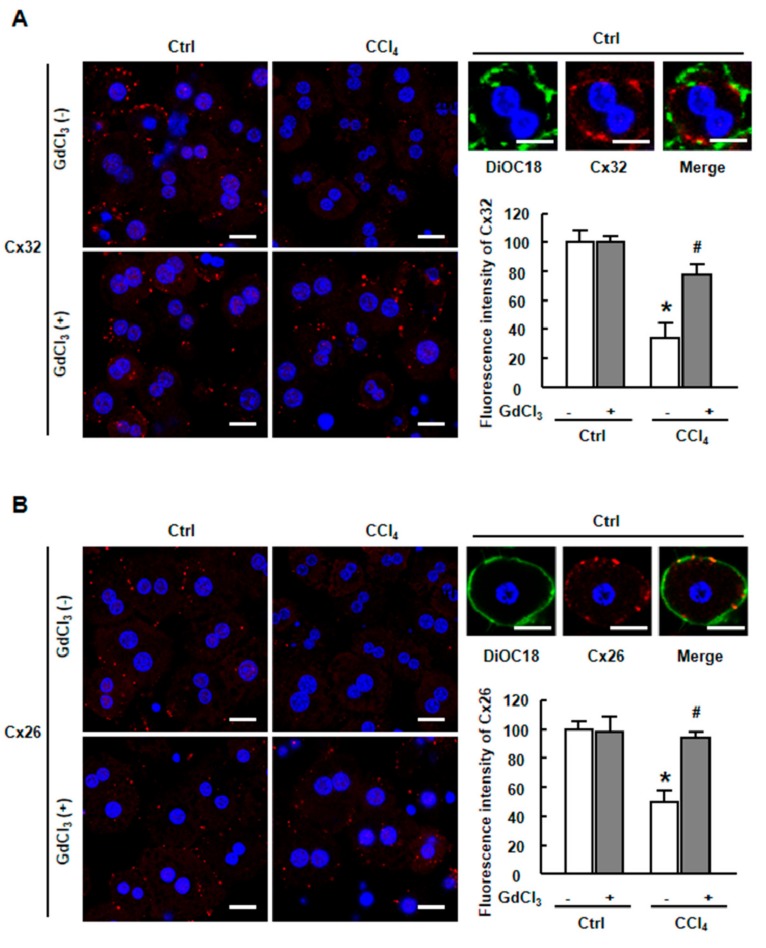
Immunofluorescence staining for Cx32 and Cx26 in CCl_4_-injured primary hepatocytes with or without GdCl_3_ pretreatment. Immunofluorescence staining for Cx32 (**A**) and Cx26 (**B**) in Ctrl-treated, CCl_4_-treated, GdCl_3_ plus CCl_4_-treated and GdCl_3_ plus CCl_4_-treated hepatocytes. Scale bars, 20 μm. The cell membrane was labeled with DiOC18, a membrane dye. Scale bars, 10 μm. DAPI was used to visualize nuclei (blue). Data are presented as the mean ± SEM, and were analyzed by ANOVA for the analysis of variance. * *p* < 0.05 vs. Ctrl. # *p* < 0.05 vs. CCl_4_-treated hepatocytes without GdCl_3_ administration.

Immunofluorescent staining for gap junction proteins, Cx32 and Cx26, was performed in primary hepatocytes to assess the effect of GdCl_3_ on connexins in vitro. Isolated hepatocytes showed a strong expression of Cx32 (Figure 6A) and Cx26 (Figure 6B) on the hepatocyte membrane under normal conditions. DiOC18, a membrane dye, was used to label this hepatocyte membrane to make sure the red stained spots of Cx32 (Figure 6A) and Cx26 (Figure 6B) were mostly placed onto the membrane surface. Importantly, the treatment of CCl_4_ resulted in the impairment of Cx32 and Cx26 plaques, which can be distinctly recovered by the administration of GdCl_3_ (Figure 6A,B). These results indicated that GdCl_3_ protected against the down-regulation of connexins and restored the Cx32/Cx26 plaques between hepatocytes in vitro.

### 2.5. GdCl_3_ Protected Hepatocytes and Contributed to the Attenuation of Liver Injury

Finally, we assessed the potential effect of GdCl_3_ on liver injury. Our previous study suggests that GdCl_3_ alleviates the extent of liver inflammation and fibrosis in CCl_4_-treated mice ^26^. Here we analyzed the protective effect of GdCl_3_ in BDL mouse models. 

**Figure 7:**
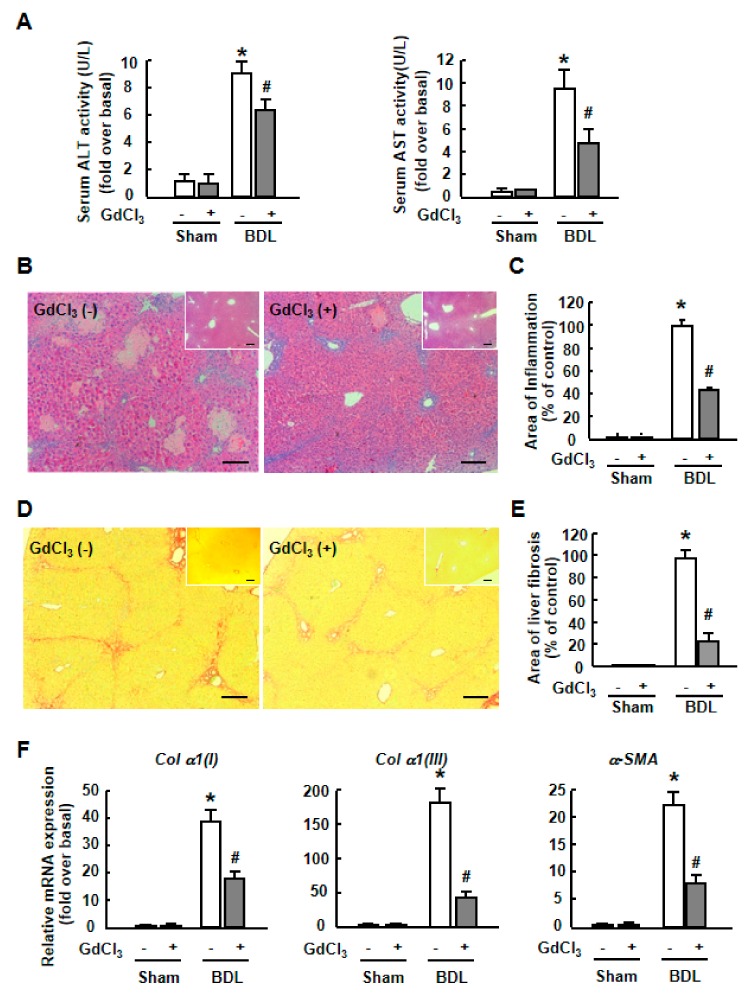
The effect of GdCl_3_ on liver inflammation and fibrosis in BDL mouse models. (**A**) Serum ALT and AST activity in BDL mice with or without GdCl_3_ administration. (**B**) Representative H&E-staining liver sections after GdCl_3_ administration in BDL liver. Scale bars, 50 µm. Inset: H&E-staining for Sham livers. (**C**) Quantification of inflammatory areas. (**D**) Representative images of Sirius Red staining after GdCl_3_ administration in BDL liver. Scale bars, 50 µm. Inset: Sirius Red staining for Sham livers. (**E**) Quantitative analysis of liver fibrosis. Ten randomly-selected fields were quantitated for each mouse using the Leica QWin V3 software. (**F**) Expression of *Col α1(I)*, *Col α1(III)*, and *α-SMA* mRNA levels in liver, measured by qRT-PCR. Data are presented as the mean ± SEM, and were analyzed by ANOVA for analysis of variance. ∗ *p* < 0.05 compared with the Sham group. # *p* < 0.05 compared with the BDL mice without GdCl_3_ administration.

As expected, the biochemical parameters indicative of liver injury, including alanine aminotransferase (ALT) and aspartate aminotransferase (AST) were proved to be decreased by GdCl_3_ in BDL mice (Figure 7A). In addition, Hematein Eosin (H&E) staining showed a significant decrease in liver injury after GdCl_3_ administration and the area of inflammation quantified by digital image analysis was dramatically reduced after GdCl_3_ administration in BDL-treated liver (Figure 7B,C). Furthermore, a diminished development of liver fibrosis was demonstrated by a reduced fibrotic area (Figure 7D,E) and the mRNA expression of fibrotic markers (*α-SMA*, *Col α1(I)*, *Col α1(III)*) (Figure 7F). Altogether, these data indicated a critical role for GdCl_3_ in chronic liver injury, showing that GdCl_3_ protected hepatocytes and attenuated liver inflammation and fibrosis in BDL mouse models.

## 3. Discussion

In the current study, we explore the critical role of GdCl_3_ in the regulation of GJIC function and connexin proteins in chronic liver injury. There are several important observations in this study. (1) Liver injury led to the dysfunction of GJIC and a down-regulation of Cx32 and Cx26 in injured liver; (2) GdCl_3_ administration markedly prevented a CCl_4_- and BDL-induced decrease of Cx32 and Cx26 at mRNA and protein levels, and restored GJIC function between hepatocytes by facilitating the transfer of the fluorescent dye from one cell into adjacent cells via GJIC in CCl_4_-treated mice; (3) CCl_4_ treatment induced a reduction of Cx32 and Cx26 mRNA and protein expression in a dose-dependent manner in primary hepatocytes, to mimic the in vivo liver injury; (4) GdCl_3_ pretreatment prevented the down-regulated levels of Cx32 and Cx26, and restored the Cx32/Cx26 plaques in CCl_4_- or LPS-injured hepatocytes; (5) In vivo GdCl_3_ administration protected hepatocytes and contributed to the attenuation of the liver injury. Altogether, these findings provide in vivo and in vitro evidence that GdCl_3_ protects functional GJIC between hepatocytes, and prevents the decrease of connexin proteins at mRNA and protein levels during liver injury, leading to the alleviation of chronic liver injury.

Much evidence has been documented supporting the hypothesis that the down-regulation of GJIC is a cellular event underlying liver disease. For example, it has been reported that Cx32 expression is lower in patients with chronic hepatitis, liver cirrhosis, and hepatocellular carcinoma than that in healthy individuals [31]. In our study, the administration of CCl_4_ or a BDL operation reduced hepatic Cx32 and Cx26 immunoreactivity and their mRNA and protein levels, which is in line with previous reports. In addition to the expression of gap junction proteins, we also examined the function of GJIC by scrape loading/dye transfer assay. Previously, Cowles et al. examined the effect of CCl_4_ on cell communication, and reported that CCl_4_ treatment did not result in the abrogation of gap junction functionality in the rat liver [17]. However, our results indicated that the transfer of Lucifer yellow dye was significantly reduced in CCl_4_-treated mouse liver, which is the first in vivo evidence to document the functional inhibition of GJIC.

Here we demonstrate for the first time that GdCl_3_ is capable of restoring and improving functional GJIC between hepatocytes in chronic liver injury. Since GdCl_3_ has been reported to be a KC-suppressing agent widely used, these changes in GJIC might be due to the actions of the proinflammatory mediators secreted by KCs. It has been reported that gap junction channel expression and/or connectivity are altered in a number of inflammatory conditions in vitro and in vivo [32]. In a spontaneous murine model of autoimmune thyroid disease, chronic inflammation reduced both Cx26 and Cx32 expression [33]. In vitro, the inhibition of GJIC by proinflammatory mediators, such as LPS, TNF-α, IL-1α or IL-1β, has been documented in different kinds of cells, including endothelial cells [34], vascular smooth muscle cells [35], Schwann cells [36], astrocytes [37] and immortalized mouse hepatocytes [38]. Furthermore, some reports have noted that proinflammatory mediators may affect connexin gene expression, the stability of connexin mRNAs or protein degradation [39,40]. Thus, it is hypothesized that the toxic agent results in the activation of KCs, which then inhibit the expression of Cx32 and Cx26, and the formation of functional hepatic GJIC between hepatocytes.

Many possible mechanisms may underlie the modification of GJIC by GdCl_3_ in chronic liver injury. For example, gadolinium has been reported to be involved in a lot of Ca^2+^-dependent physiologic processes due to its biophysical similarities to calcium [41]. 

In our study, we also examined the effects of Mg^2+^ and Ca^2+^ upon the expression of connexin proteins in CCl_4_- or LPS-treated hepatocytes, showing that MgCl_2_ or CaCl_2_ had no influence on the mRNA expression of Cx32 and Cx26. Moreover, several studies suggest that gadolinium serves as an alleviator of P450 induction [42] and an opener of plasma-integrated porin channels [43]. Further studies will be needed to assess the exact mechanism underlying the regulation of GJIC function and the expression of Cx32 and Cx26 by GdCl_3_ in our models. 

Numerous studies have documented that GdCl_3_ contributed to the attenuation of liver injury. For instance, GdCl_3_ could partially attenuate binge drinking-induced liver steatosis, which might be attributed to the suppression of the mobilization of white adipose tissues [44]. Our previous study also suggested that GdCl_3_ alleviated the extent of liver inflammation and fibrosis in CCl_4_-treated mice, as demonstrated by the reduced expression of fibrotic markers and the decreased inflammatory and fibrotic area using H&E and Sirius Red staining [26]. Here we demonstrated that the improvement of GJIC function and connexin expression by GdCl_3_ was beneficial to the amelioration of hepatic fibrogenesis in BDL mice. In fact, the restoration of GJIC by targeting connexin expression is a very attractive anticancer strategy [45]. Sai and Kang have reported that green tea or its major component epicatechin might prevent mouse hepatocarcinogenesis via its ability to prevent the down-regulation of GJIC [46,47]. Thus, the improvement of GJIC function by GdCl_3_ might be a promising anti-inflammatory and anti-fibrotic strategy, which will have a significant clinical impact.

In summary, the present study provides evidence that GdCl_3_ protects against the dysfunction of GJIC and restores the reduced expression of connexin proteins, Cx32 and Cx26 between hepatocytes in chronic liver injury mice and primary hepatocytes, which provides new insights into the effects of GdCl_3_ during liver injury.

## 4. Materials and Methods

### 4.1. Materials

Antibodies against Cx32 and Cx26 were purchased from Sigma (St. Louis, MO, USA) and Invitrogen (Camarillo, CA, USA), respectively. Cy3-conjugated AffiniPure goat anti-rabbit IgG antibody came from Jackson ImmunoResearch Laboratories Inc. (West Grove, PA, USA). Lucifer yellow dye was obtained from Invitrogen (Eugene, OR, USA). Gadolinium chloride hexahydrate and other reagents were from Sigma (St. Louis, MO, USA).

### 4.2. Mouse Models of Liver Injury

To induce the carbon tetrachloride (CCl_4_)-induced liver injury model, mice received intraperitoneal injections of 1 μL per gram body weight (B.W.) of a CCl_4_/olive oil (OO) mixture (1:9 v/v) twice per week, and were sacrificed at day 1, 2, 7 or at 28 days. A bile duct ligation (BDL) operation was performed as described previously [48]. Clodronate liposome (10 μL/g B.W., YeaSen, Shanghai, China) injection was performed once, 72 hours before CCl_4_ treatment, in order to delete Kupffer cells. Gadolinium chloride (GdCl_3_) (7.5 mg/kg B.W.) or saline was administered intraperitoneally the day before the CCl_4_ treatment or BDL operation. These mice were sacrificed at 28 days of CCl_4_ treatment or at 14 days of BDL. Liver tissue and blood samples were collected. All animal work was conformed to the Ethics Committee of Capital Medical University, and in accordance with the approved guidelines (Animal Experiments and Experimental Animal Welfare Committee of Capital Medical University, approval number: AEEI-2014-131, project identification code: 0100501017, 27 December 2014).

### 4.3. Mouse Primary Hepatocyte Isolation and Culture

To isolate primary murine hepatocytes, anesthetized and heparinized mice were subjected to a midline laparotomy and cannulation of the portal vein, followed by liver perfusion with an EGTA-chelating perfusion buffer. After perfusion with a 0.4% collagenase buffer, their livers were minced and cells dispersed in culture medium; hepatocytes and nonparenchymal cells were separated using low-speed centrifugation and 40% percoll density gradient centrifugation. Isolated mouse hepatocytes (2 × 10^5^ /well) were cultured in William’s Medium E (Gibco, Life Technologies, Foster City, CA) with 10% FBS on 24-well collagen-coated plate at 37 °C with 5% CO_2_ for 4 h.

Hepatocytes were incubated with the pre-treatment of GdCl_3_, MgCl_2_ or CaCl_2_, followed by CCl_4_ or lipopolysaccharide (LPS) treatment. After two hours of culture, the cells were used for immunofluorescence staining, real-time reverse transcription polymerase chain reaction (RT-PCR) and western blot.

### 4.4. Real-time RT-PCR

Total RNA was extracted from liver frozen specimens, as described previously using an RNeasy kit (Qiagen, Hilden, Germany). Real-time RT-PCR was performed in an ABI Prism 7300 sequence detecting system (Applied Biosystems, Carlsbad, CA, USA), as described previously [48]. Primers used for real-time RT-PCR were as follows: 18S rRNA: sense, 5′-GTAACCCGTTGAACCCCATT-3′; antisense, 5′-CCATCCAATCGGTAGTAGCG-3′. Mouse Cx32: sense, 5′-AAACCGTCTTCACTGTCTTTATGCT-3′; anti-sense, 5′-CCGCCACGTTGAGGATAATG-3′. Cx26: sense, 5′-TCATGGGTTTGCTTGGGAAT-3′; anti-sense, 5′-CCATTTGGTTTCTGCACCATT-3′. Cx30.3: sense, 5′-GTGTGGGACGACGATCAAAAG-3′; anti-sense, 5′-TGACCACTAACAGGGAAGGAC-3′. Cx37: sense, 5′-CCCACATCCGATACTGGGTG-3′; anti-sense, 5′-CGAAGACGACCGTCCTCTG-3′. Cx30.2: sense, 5′-TCATGCTGATCTTCCGCATCC-3′; anti-sense, 5′-GAAGCGGTAGTGGGACACC-3′. Cx29: sense, 5′-GAAGGATGTGTTAAGCCTCCAA-3′; anti-sense, 5′-CTCATTCCCGTAGACAGCAAAG-3′. Cx47: sense, 5′-TCCACAATCATTCCACCTTCG-3′; anti-sense, 5′-CAGAAGCGCACATGAGACAG-3′. Cx46: sense, 5′-CACAGGAGCACTCTACAGTCA-3′; anti-sense, 5′-CGGTCGTAGCAGACGTTCTC-3′. Cx31: sense, 5′-GCTCCAAGACCTATTGAGTGGC-3′; anti-sense, 5′-GCCTGGTGTTACAGTCAAAGTC-3′. Cx31.1: sense, 5′-TGTGGGGAGACGACCAGAA-3′; anti-sense, 5′-CGGGATTCGGGTAAAGGTAAC-3′. Cx39: sense, 5′-ATCTGGCTGATCGTGGAGGT-3′; anti-sense, 5′-GGGAAAAGAGGTCGTAGCAAA-3′. Cx36: sense, 5′-ATGGGGGAATGGACCATCTTG-3′; anti-sense, 5′-TCATCATCGTACACCGTCTCC-3′. Cx45: sense, 5′-CAGAGCCAACCAAAACCTAAGC-3′; anti-sense, 5′-CTGCACACATAAAATGGGTGGA-3′. Cx40: sense, 5′-GGTCCACAAGCACTCCACAG-3′; anti-sense, 5′-CTGAATGGTATCGCACCGGAA-3′. Cx30: sense, 5′-ACCAGCATAGGGAAGGTGTG-3′; anti-sense, 5′-TGCAGAGTGTTGCAGACAAAG-3′. Cx50: sense, 5′-AATGAGCACTCCACTGTCATCG-3′; anti-sense, 5′-TGGGTGTTGCATACAAAATCAGA-3′. Cx43: sense, 5′-TGTGCCCACACTCCTGTACTTG-3′; anti-sense, 5′-TTTCTTGTTCAGCTTCTCTTCCTTT-3′. Cx23: sense, 5′-TGCTGTCTACGGGAATGAGG-3′; anti-sense, 5′-CCGGAACTGATTGTAACAGAACA-3′. Cx33: sense, 5′-ATGAGTGATTGGAGTGCCTTACA-3′; anti-sense, 5′-CAAGCCGACTCGATA GCAGTG-3′. Cx57: sense, 5′-AATTTACTGGGTGGCATCCTAGA-3′; anti-sense, 5′-GGGAAAGCATCATCGTAACAGAT-3′. α-SMA: sense, 5′-ATGCTCCCAGGGCTGTTTT-3′; anti-sense, 5′-TTCCAACCATTACTCCCTGATGT-3′. Col α1(I): sense, 5′-AGGGCGAGTGCTGTGCTTT-3′; anti-sense, 5′-CCCTCGACTCCTACATCTTCTGA-3′. Col α1(III): sense, 5′-TGAAACCCCAGCAAAACAAAA-3′; anti-sense, 5′-TCACTTGCACTGGTTGATAAGATTAA-3′. 

### 4.5. Immunofluorescence Staining

Liver samples were fixed in 4% paraformaldehyde and embedded in Tissue Tek OCT compound (Sakura Finetek USA, Inc., CA, USA); 7 μm frozen sections were cut and blocked with 2% bovine serum albumin in PBS for one hour, and then incubated with anti-Cx32 (1:400) or anti-Cx26 (1:100) antibody. Cy3-conjugated AffiniPure goat anti-rabbit IgG antibody (1:100) was used as a secondary antibody. The sections were covered with Vectashield mounting medium containing 4,6-diamidino-2-phenylindole and observed under a confocal microscope (LEICA TCS SP5, LASAF 2.5, Wetzlar, Germany).

### 4.6. Quantitative Analysis of Liver Fibrosis and Inflammation

Liver paraffin sections (5 µm) were stained with Sirius red for collagen visualization and H&E for analysis of the inflammatory area. The fibrotic area and inflammatory area were assessed by computer-assisted image analysis with MetaMorph software (Universal Imaging Corporation, Downingtown, PA, USA) as described [48]. The mean value of 15 randomly-selected areas per sample was used as the expressed percentage of the fibrosis or necrosis area.

### 4.7. Scrape Loading/Dye Transfer assay

The function of gap junction intercellular communication was measured by scrape loading/dye transfer assay as follows. 5 mm-thick liver slices were cut 3 to 4 incisions (1 mm in depth) with a blade, followed by the dropping of 0.05% Lucifer yellow on the liver slices. After 3 min, the slices were washed in PBS three times, then embedded and frozen in the Tissue Tek OCT compound. Seven μm-thick frozen sections were made and photographed by a fluorescence microscope. Spreading of the dye was measured using an image analyzer (Leica Qwin V3 software, Wetzlar, Germany). 

### 4.8. Western Blot Analysis

Western blot analysis of Cx32 and Cx26 was performed with 50 μg of protein extract, obtained as described previously [48] using rabbit polyclonal antibody to Cx32 (1:500) or Cx26 (1:1000). Peroxidase-conjugated goat anti-rabbit IgG antibody (1:5000) was used as a secondary antibody. Protein expression was visualized by using an enhanced chemiluminescence (ECL Plus) assay kit according to the manufacturer’s instructions (Amersham Biosciences, Arlington Heights, IL, USA). The bands were quantified using the GeneSnap and GeneTools software from PerkinElmer (Waltham, MA, USA), and the results were normalized relative to tubulin (rabbit monoclonal anti-tubulin antibody, 1:1000, Epitomics, Burlingame, CA, USA) expression to correct for variations in protein loading and transfer.

### 4.9. ALT and AST Quantification

The ALT and AST levels were detected by BS-200 Chemistry Analyzer (Mindray, Shenzhen, China).

### 4.10. Statistics

These results are expressed as mean ± SEM, and were analyzed by Student’s t-test (when two groups were compared) or ANOVA (when three or more groups were compared) for an analysis of variance (*p* < 0.05 was considered significant).
